# Outcomes Assessment of Hypospadias Repair

**DOI:** 10.7759/cureus.48808

**Published:** 2023-11-14

**Authors:** Jayaditya Devpal Patil, Yusuf Mahdi Mohamed, Abeer Farhan, Martin Corbally

**Affiliations:** 1 Department of Medicine, Royal College of Surgeons in Ireland - Medical University of Bahrain, Busaiteen, BHR; 2 Department of Surgery, King Hamad University Hospital, Busaiteen, BHR; 3 Department of Surgery, Royal College of Surgeons in Ireland - Medical University of Bahrain, Busaiteen, BHR

**Keywords:** surgical repair, functional outcomes, pediatric, cosmetic outcome, hypospadias repair

## Abstract

Introduction: Hypospadias is a congenital malformation, which involves the displacement of the urethral orifice on the underside of the penis. The mainstay treatment of hypospadias is surgery. Currently, there is no literature broadly assessing hypospadias repair outcomes in the Kingdom of Bahrain. This study aims to provide descriptive data on cosmetic, functional, and surgical outcomes of hypospadias repair at a single medical institute in the Kingdom of Bahrain.

Method: Data on patients who underwent hypospadias repair from January 2012 to December 2020 by a single surgeon were reviewed. Parents of patients were contacted via telephone for consent. All consenting participants returned for an outpatient assessment of functional and cosmetic outcomes using an original questionnaire and the Pediatric Penile Perception Score, respectively. All responses were recorded using a four-point Likert scale. Surgical outcomes were assessed by reviewing postoperative notes. All collected data were anonymized. The study was approved by the King Hamad University Hospital institutional review board.

Results: Of the 29 patients who underwent surgical repair for hypospadias, 15 patients consented to participate. The mean age of the study population was 2.466 (SD = 0.496). Both parent and physician cosmetic assessments had similar results with the majority of participants very satisfied with all cosmetic parameters. Physician assessment reported higher satisfaction compared to parents. There were no reported cases of poor satisfaction. In terms of functional outcomes, there were no reported cases of straining on initiation, and a smooth and continuous urinary stream was reported in 80%. Only four patients reported post-void dribbling. When assessing surgical outcomes, 53.30% had coronally located urethra with no cases of postoperative complications. More than half of our patients were discharged after one day.

Conclusion: Our study noted overall high satisfaction in terms of surgical, cosmetic, and functional outcomes. Physicians reported better cosmetic outcomes when compared to parents. Further analysis with a larger sample size across various medical institutes will be required to better assess post-repair outcomes.

## Introduction

Hypospadias is a congenital malformation, which involves the displacement of the urethral orifice on the underside of the penis. The severity of the condition is often classified according to factors such as the anatomical location of the urethral orifice along the penile shaft, degree of penile curvature, and appearance of the foreskin [1,2]. In current practice, the mainstay management of hypospadias is surgery, and with an incidence of one in 200 live male births, surgical correction of hypospadias is a very commonly performed procedure [3,4]. The surgical technique used for correction largely depends on the severity of the condition, clinician experience, and family preferences. Examples of such procedures include a tubularized incision plate, an onlay island flap for more severe forms of hypospadias, and correction of penile curvature [5]. There have been numerous studies evaluating the surgical outcomes of various types of hypospadias repair procedures, most indicating low postoperative complications [6]. Much of the available literature is limited by short follow-up periods. High-quality randomized trials are extremely challenging and rarely performed [1,6,7]. The implications of hypospadias to the affected child include abnormal voiding patterns, potential sexual dysfunction, psychological impact, and the prospect of surgical procedures as a child. Despite these significant cosmetic and functional sequelae, there is little published data in the current literature regarding different outcomes of hypospadias repair. A common area of weakness of the existing literature is that most studies have been exclusively based on surgeon impressions of the repair, without considering patient or parent satisfaction [8,9]. Additionally, significant discrepancies exist in the current literature, where some studies report normal sexual development and function while others report fewer positive results [10-13]. The Pediatric Penile Perception Score (PPPS) is a validity tool that was proposed to overcome this form of observer bias [10]. To our knowledge, there is no Bahrain-based literature on this subject, including studies performed at our institute, the King Hamad University Hospital (KHUH). Only one study related to hypospadias has been published, evaluating the general knowledge of the condition amongst health practitioners in the Kingdom of Bahrain [14]. The lack of local descriptive data on the surgical, functional, and cosmetic outcomes is a limitation for surgeons, as continuous clinical data are crucial for the assessment of procedural efficacy along with overall prognosis. The discrepancies among functional and cosmetic outcomes also prompted us to further investigate these outcomes at KHUH, Bahrain. This study aims to provide descriptive data on the surgical, functional, and cosmetic outcomes of hypospadias repair to increase awareness among pediatricians and surgeons across various medical institutes in the Kingdom of Bahrain.

This article was previously presented as a poster at the 2023 Royal College of Surgeons in Ireland Annual Research Conference on March 6, 2023.

## Materials and methods

This study was conducted in five stages: patient chart review, patient contact identification, telephone verbal consent, outpatient cosmetic and functional assessment, and postoperative data analysis. The study was approved by the KHUH Institutional Review Board (approval number: 21-425). Over the course of nine years, all charts for patients, regardless of age, who underwent surgical hypospadias repair at KHUH by a single pediatric surgeon, from January 2012 to December 2020, were reviewed from the hospital's electronic software. Data retrieved included parent demographics, contact details, and surgical procedures. All collected data were anonymized to avoid patient identifiers. All eligible candidates were later contacted via telephone over a two-week period during which details of the study and participant responsibilities were shared. Informed verbal consent was obtained from parents for the participation of their child in the study. Parents who refused consent were not included in the study and their data were not reviewed. All consenting parents returned for an outpatient appointment at the pediatric department in KHUH to assess functional and cosmetic outcomes. All outpatient consultations were conducted with a qualified pediatric senior house officer and two additional research team members. Functional outcomes were assessed using an original questionnaire while cosmetic outcomes were measured using the PPPS. The original questionnaire was generated by the research team, involving the operating surgeon, with the aim of assessing straining on initiation, spraying during urination, smoothness and continuity of urine stream, post-void dribbling, and straightness of the penis during erection. All responses were classified as either yes or no. The PPPS comprised of five cosmetic components: shaft skin appearance, meatal position and configuration, glans position and configuration, penile axis, and general penile appearance. Responses were measured using a four-point Likert scale ranging from zero to three, with the lowest value "zero" resembling very dissatisfied and “three” resembling very satisfied. The PPPS is comprised of feedback from the patient, parent, and doctor. In our study, patient self-assessment was not measured as participants were not old enough to comprehend the scoring system or accurately self-assess. Surgical outcomes were measured using postoperative case notes. Data retrieved included patient age at the time of surgery, surgical technique, blood loss, postoperative infection rates within the first week, length of postoperative stay, patient satisfaction, meatal stenosis, urethrocutaneous fistula, dehiscence, and urethral diverticulum. All participant responses were encrypted and stored on the research team’s computer.

## Results

Of the 29 patients who underwent surgical intervention for hypospadias repair at KHUH from January 2012 to December 2020, only 15 parents consented on behalf of their child to participate in our study. Patient characteristics of non-participating patients were not reviewed for the purpose of this study. The sample age of patients upon review ranged from one to seven years old with a mean age of 2.466 (SD: 0.496) years across the study group. Our study analyzed three categories of outcomes: cosmetic, functional, and surgical.

Cosmetic outcomes

The physician assessment was implemented by a qualified pediatric senior house officer at KHUH. Cosmetic parameters measured included penile shaft appearance, meatal and glans position, penile configuration, penile axis and mucosal collar, and general penile appearance. Physician PPPS assessment is summarized in Table 1.

**Table 1:** Doctor Pediatric Penile Perception Score (PPPS) response frequency

	Level of satisfaction of doctor PPPS assessment, (n) %, Likert scale (0-3)				
Cosmetic variables	Very dissatisfied (0)	Dissatisfied (1)	Satisfied (2)	Very satisfied (3)	Total sample size (n)
Shaft skin appearance	0 (0.00%)	0 (0.00%)	2 (13.30%)	13 (86.70%)	15
Meatal position and configuration	0 (0.00%)	0 (0.00%)	2 (13.30%)	13 (86.70%)	15
Glans position and configuration	0 (0.00%)	0 (0.00%)	2 (13.30%)	13 (86.70%)	15
Penile axis and mucosal collar	0 (0.00%)	0 (0.00%)	1 (6.70%)	14 (93.30%)	15
General penis appearance	0 (0.00%)	0 (0.00%)	1 (6.70%)	14 (93.30%)	15

Parent PPPS scores were reported as a mutual agreement between both parents of the patient whenever applicable. Parent PPPS assessment is depicted in Table 2.

**Table 2:** Parent Pediatric Penile Perception Score (PPPS) response frequency

	Level of satisfaction of parent PPPS assessment, (n) %, Likert scale (0-3)				
Cosmetic variables	Very dissatisfied (0)	Dissatisfied (1)	Satisfied (2)	Very satisfied (3)	Total sample size (n)
Shaft skin appearance	0 (0.00%)	0 (0.00%)	6 (40.00%)	9 (60.00%)	15
Meatal position and configuration	0 (0.00%)	0 (0.00%)	7 (46.70%)	8 (53.30%)	15
Glans position and configuration	0 (0.00%)	0 (0.00%)	6 (40.00%)	9 (60.00%)	15
Penile axis and mucosal collar	0 (0.00%)	0 (0.00%)	6 (40.00%)	9 (60.00%)	15
General penis appearance	0 (0.00%)	0 (0.00%)	6 (40.00%)	9 (60.00%)	15

Functional outcomes

Functional outcomes were assessed using an original questionnaire generated by the research team. All assessments were made by the parents and all parameters received 100% response apart from the assessment of a straight penis during erection, where 14 parents responded, of which only 12 parents reported a straight penis on erection. All parents reported no straining on initiation. Spraying on urination was reported in only three patients. A smooth and continuous urinary stream was reported in 80%. Post-void dribbling was reported in only four patients.

Surgical outcomes

**Figure 1:**
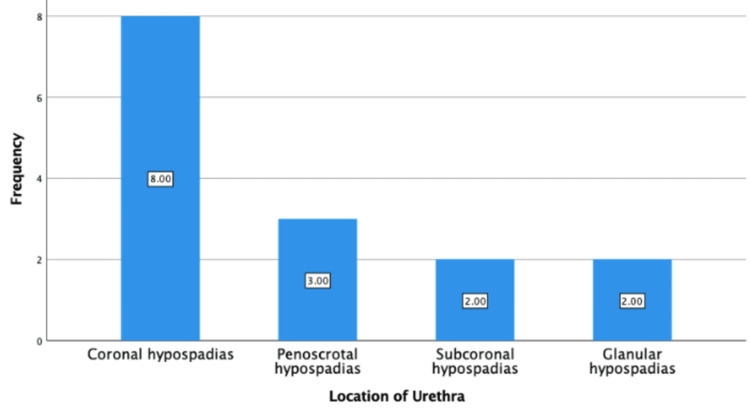
Frequency of preoperative location of urethra

All 15 cases of hypospadias had varying urethral anatomy prior to surgery (Figure 1). More than half of the patients had a coronal subtype. Postoperatively, none of our patients reported meatal stenosis, urethrocutaneous fistula, dehiscence, urethral diverticulum, or postoperative infection. In terms of postoperative stay, more than half of our patients were discharged after one day.

## Discussion

This descriptive study aimed to obtain data on functional, cosmetic, and surgical outcomes in patients who underwent hypospadias repair at KHUH. Cosmetic assessment is usually performed by the operating surgeon; however, such practice leads to inaccuracy and subjectiveness. The PPPS is a reliable tool for objectively assessing cosmetic outcomes [15]. It is one of very few tools that takes patient self-assessment into account. This is important as studies in the past have suggested psychosexual health to be affected in patients with hypospadias, with some suggesting patient self-assessment to play a key role [16,17]. Parents' attitudes toward their child's genital appearance may also influence the patient's genital perception and development [18]. These factors collectively support the incentive to use the PPPS in our study. Our results found that across all parameters, physician PPPS reported higher satisfaction rates than parent assessment. Although these findings support good overall cosmetic outcomes, further investigation into lower parent satisfaction rates may be warranted, where managing postoperative cosmetic expectations may play a role in improving overall satisfaction. The PPPS implementation in our study, however, did not record patient self-assessment. The rationale behind this being underage patients and their inability to comprehend and accurately use the scoring system.

Functional outcomes were assessed using an originally constructed questionnaire reviewing various aspects of penile and urinary function. Our study reported very good outcomes in terms of straining on initiation and spraying and smoothness of the urinary stream. However, a few cases did suggest some deficit in terms of post-void dribbling. One study reported that patients with hypospadias show abnormal flow patterns pre- and postoperatively. This may explain the few discrepancies in functional outcomes in our study; however, urodynamic studies will need to be performed to correlate with the clinical findings [19,20]. Although our study highlighted significant results, sexual function could not be assessed due to patients' age. Engaging in conversations regarding sexual behavior is a sensitive topic in regions in the Middle East. However, its significance has been extensively reported in cases of hypospadias repair. Studies have shown patients who underwent hypospadias surgery are concerned about penile appearance and the more severe the hypospadias, the more dissatisfactory the long-term outcome. Data have also highlighted a relatively high incidence of erectile dysfunction and premature ejaculation [21-23]. Our study may benefit by conducting a long-term, multi-center follow-up, which would allow us to accurately assess sexual outcomes.

Continual evaluation of postoperative outcomes is vital in the surgical management of any pathology. This is particularly the case for hypospadias repair where cosmetic outcomes need to be strongly considered during surgical intervention. The most common complications following hypospadias repair are urethrocutaneous fistula, meatal stenosis, urethral stricture, urethral diverticulum, glans dehiscence, breakdown, and unfavorable cosmetic outcomes requiring redo surgery [2]. Our study reported no cases of such complications. Additionally, no cases of postoperative infections were recorded incurring good overall surgical outcomes. To further improve management, various outcomes of hypospadias repair need to be reported and assessed at pre-determined time intervals. As of now, there are no established postoperative follow-up guidelines for hypospadias repair. Some studies suggest most complications present within a short period postoperatively and hence consider follow-up for six months to be adequate [24]. However, some data have shown good long-term outcomes without further complications in 75% of the patients. Of the remaining 25% who required re-operation, only 47.37% presented in the first 12 months postoperatively [25]. This highlights the need for a structured long-term follow-up plan post-hypospadias repair. Additionally, there is limited data on the incidence of failed hypospadias repair in childhood and no reliable estimate of the number of patients undergoing further surgery in adulthood [26].

Limitations and strengths

Our study was met with limitations, particularly with a lack of consistency in data storage and difficulty with data extraction. Our study did not adhere to a structured follow-up time frame as patients were recalled over a nine-year period, regardless of elapsed time postoperatively. We reviewed patients who had undergone hypospadias repair by a single surgeon to reduce bias. However, this was met with a limited sample size. Although 25 patients were contacted, only 15 consented to participation. Despite drawbacks, our study was able to accurately assess functional and cosmetic outcomes using a universal assessment tool. A larger multi-center study with a structured follow-up plan and inclusion of numerous operating surgeons would allow for a more accurate assessment of various post-hypospadias repair outcomes.

## Conclusions

Our study concludes patients who have undergone hypospadias repair at KHUH have overall good cosmetic, functional, and surgical outcomes. Physicians reported better cosmetic outcomes when compared to parents. A larger, multi-center study assessing sexual outcomes as well as patient self-assessment is warranted to provide a better understanding of the outcomes of hypospadias repair in the Kingdom of Bahrain.

## Appendices

**Table 3:** Original questionnaire for the assessment of functional outcomes

Functional assessment	Yes	No
Straining on initiation		
Spraying during urination		
Smoothness and continuity of urine stream		
Post-void dribbling		
Straightness of the penis during erection		

## References

[1] van der Horst HJ, de Wall LL (2017). Hypospadias, all there is to know. Eur J Pediatr.

[2] Springer A (2014). Assessment of outcome in hypospadias surgery - a review. Front Pediatr.

[3] Nelson CP, Park JM, Wan J, Bloom DA, Dunn RL, Wei JT (2005). The increasing incidence of congenital penile anomalies in the United States. J Urol.

[4] Schnack TH, Poulsen G, Myrup C, Wohlfahrt J, Melbye M (2010). Familial coaggregation of cryptorchidism, hypospadias, and testicular germ cell cancer: a nationwide cohort study. J Natl Cancer Inst.

[5] Baskin LS, Wilcox D (2022). Hypospadias: management and outcome. UpToDate.

[6] Wang F, Xu Y, Zhong H (2013). Systematic review and meta-analysis of studies comparing the perimeatal-based flap and tubularized incised-plate techniques for primary hypospadias repair. Pediatr Surg Int.

[7] Tekgül S, Riedmiller H, Hoebeke P (2012). EAU guidelines on vesicoureteral reflux in children. Eur Urol.

[8] Jayanthi VR, McLorie GA, Khoury AE, Churchill BM (1995). Functional characteristics of the reconstructed neourethra after island flap urethroplasty. J Urol.

[9] Garibay JT, Reid C, Gonzalez R (1995). Functional evaluation of the results of hypospadias surgery with uroflowmetry. J Urol.

[10] Mureau MA, Slijper FM, Slob AK, Verhulst FC, Nijman RJ (1996). Satisfaction with penile appearance after hypospadias surgery: the patient and surgeon view. J Urol.

[11] Sommerlad BC (1975). A long-term follow-up of hypospadias patients. Br J Plast Surg.

[12] Avellán L (1976). The development of puberty, the sexual début and sexual function in hypospadiacs. Scand J Plast Reconstr Surg.

[13] Berg R, Svensson J, Astrom G (1981). Social and sexual adjustment of men operated for hypospadias during childhood: a controlled study. J Urol.

[14] Al Rashed A, Mubarak M, Abbas M, Singal AK (2019). Physicians approach to hypospadias in Kingdom of Bahrain. J Bah Med Soc.

[15] Weber DM, Schönbucher VB, Landolt MA, Gobet R (2008). The Pediatric Penile Perception Score: an instrument for patient self-assessment and surgeon evaluation after hypospadias repair. J Urol.

[16] Mureau MA, Slijper FM, Nijman RJ, van der Meulen JC, Verhulst FC, Slob AK (1995). Psychosexual adjustment of children and adolescents after different types of hypospadias surgery: a norm-related study. J Urol.

[17] Schönbucher VB, Weber DM, Landolt MA (2008). Psychosocial adjustment, health-related quality of life, and psychosexual development of boys with hypospadias: a systematic review. J Pediatr Psychol.

[18] Schultz JR, Klykylo WM, Wacksman J (1983). Timing of elective hypospadias repair in children. Pediatrics.

[19] Perera M, Jones B, O'Brien M, Hutson JM (2012). Long-term urethral function measured by uroflowmetry after hypospadias surgery: comparison with an age matched control. J Urol.

[20] Wolffenbuttel KP, Wondergem N, Hoefnagels JJS (2006). Abnormal urine flow in boys with distal hypospadias before and after correction. J Urol.

[21] Jiao C, Wu R, Xu X, Yu Q (2011). Long-term outcome of penile appearance and sexual function after hypospadias repairs: situation and relation. Int Urol Nephrol.

[22] Rynja SP, Wouters GA, Van Schaijk M, Kok ET, De Jong TP, De Kort LM (2009). Long-term followup of hypospadias: functional and cosmetic results. J Urol.

[23] Chertin B, Natsheh A, Ben-Zion I, Prat D, Kocherov S, Farkas A, Shenfeld OZ (2013). Objective and subjective sexual outcomes in adult patients after hypospadias repair performed in childhood. J Urol.

[24] Snodgrass W, Macedo A, Hoebeke P, Mouriquand PD (2011). Hypospadias dilemmas: a round table. J Pediatr Urol.

[25] Spinoit AF, Poelaert F, Groen LA, Van Laecke E, Hoebeke P (2013). Hypospadias repair at a tertiary care center: long-term followup is mandatory to determine the real complication rate. J Urol.

[26] Barbagli G, Perovic S, Djinovic R, Sansalone S, Lazzeri M (2010). Retrospective descriptive analysis of 1,176 patients with failed hypospadias repair. J Urol.

